# Dengue Incidence, Seroprevalence, and Expansion Factors from Active Surveillance, Brazil, 2016–2021

**DOI:** 10.3201/eid3204.250942

**Published:** 2026-04

**Authors:** Eliana Nogueira Castro de Barros, Manuela de Almeida Roediger, Maïna L’Azou Jackson, Morgan A. Marks, Elizabeth M. Anderson, Alejandra Esteves-Jaramillo, Germán Áñez, José A. Sousa Moreira, Fernanda Castro Boulos

**Affiliations:** Instituto Butantan, São Paulo, Brazil (E.N.C. Barros, M.A. Roediger, J.A.S. Moreira, F.C. Boulos); MSD (UK) Limited, London, UK (M. L'Azou Jackson); Merck & Co., Inc., Rahway, New Jersey, USA (M.A. Marks, E.M. Anderson, A. Esteves-Jaramillo, G. Áñez)

**Keywords:** arbovirus, dengue, viruses, seroprevalence, surveillance, epidemiology, chikungunya, vector-borne infections, Brazil

## Abstract

Dengue is hyperendemic in Brazil and is underestimated by passive surveillance. To better understand dengue incidence, we conducted epidemiologic analyses among participants, 2–59 years of age, from the placebo arm of a phase 3 dengue vaccine trial. During 2016–2021, a total of 5,947 participants contributed to 22,028 person-years of follow-up. We identified and virologically confirmed dengue (VCD), Zika, and chikungunya infections. We observed VCD and chikungunya incidence heterogeneity by age, geographic location, and study year. Children 2–6 years of age experienced the highest VCD (2.33/100 person-years) and chikungunya (1.02/100 person-years) incidence. VCD peaked in 2019 (n = 148) whereas chikungunya peaked in 2017 (n = 51). VCD incidence rates from active surveillance were generally higher than those reported to the national passive surveillance system; expansion factor range was <1–9.5 by municipality. Active surveillance is critical to better understand and characterize dengue epidemiology.

Dengue, caused by infection with one of 4 dengue virus (DENV) serotypes, is endemic in over 100 countries (*1*–*4*). Clinical manifestations of dengue range from asymptomatic to severe illness that can be fatal. Dengue is the most rapidly spreading mosquitoborne viral disease, increasing faster than any other communicable disease (*5*). Dengue mainly circulates in tropical and subtropical climates; most cases occur in Southeast Asia and Latin America (*2*,*4*). However, cases outside endemic areas are becoming more frequent because of increases in urbanization, travel to endemic regions, and the expansion of the primary vector (*6*,*7*).

More than 50% of the dengue cases in Latin America are reported in Brazil (*8*,*9*). In a 2024 outbreak, >10 million cases and >6,000 deaths were reported across Brazil (*10*,*11*). All 4 DENV serotypes have been reported in Brazil since 2010; typically, 2 serotypes cocirculate with variations in the dominant serotype over time (*10*). In addition to DENV, chikungunya virus (CHIKV) and Zika virus (ZIKV), which are transmitted by the same *Aedes* spp. mosquito vector, circulate in Brazil (*12*). CHIKV was first identified in Brazil in 2014 and causes annual epidemics (*13*,*14*). In 2015, a major outbreak of Zika led the Brazil Ministry of Health to declare a national health emergency (*15*,*16*).

The epidemiology of dengue in Brazil has previously been described using data collected through passive surveillance (*17*). Dengue, chikungunya, and Zika are notifiable diseases in Brazil; healthcare institutions report suspected, probable, and laboratory-confirmed cases, along with basic demographic information, to the national passive surveillance system, Sistema de Informação de Agravos de Notificação (SINAN) (*17*). However, because of the subclinical manifestation of dengue, many patients do not seek medical care. In addition, the nonspecific clinical presentation that overlaps with other arboviral diseases, including chikungunya and Zika, can cause misclassification. Those factors, along with reporting biases, can result in an underestimation of dengue burden by passive surveillance systems (*18*). Instead, active surveillance of prospective longitudinal cohorts can better estimate the burden of dengue with generally higher incidence rates than those reported by the national surveillance system (*19**,**20*). 

To better understand dengue in Brazil, we conducted epidemiologic analyses among participants enrolled in a large phase 3 efficacy trial of a live attenuated tetravalent dengue vaccine, Butantan-DV (*21**,**22*). We described dengue serostatus by demographic characteristics before receipt of vaccine or placebo. We used the active surveillance of participants in the placebo arm of the trial to estimate incidence rates of dengue during 2016–2021 and determine the degree of underreporting compared with the national passive surveillance system.

## Materials and Methods

### Dengue Vaccine Efficacy Trial Design and Participants

DEN-03-IB (clinical trials identifier NCT02406729) is a double-blind, randomized, placebo-controlled, phase 3 trial to assess the safety and efficacy of a single dose of Butantan-dengue vaccine (Butantan-DV) for the prevention of symptomatic virologically confirmed dengue (VCD) (*21**,**22*). Participants included healthy persons 2–59 years of age who were enrolled and randomized 2:1 (Butantan-DV:placebo), stratified by age group (2–6, 7–17, and 18–59 years), at 16 sites in 15 municipalities across the 5 geographic regions of Brazil; 3 sites in the North, 4 sites in the Northeast, 3 sites in the Center-West, 5 sites in the Southeast, and 1 site in the South region. National Commission for Research Ethics (approval no. CAAE 44462915.8.1001.0068) and local ethics committees approved the study; it was conducted in accordance with the principles of good clinical practice.

### Seropositivity Measurements

We determined baseline dengue serostatus retrospectively using blood samples collected on day 0 from enrolled participants, before receipt of vaccine or placebo. Samples were tested in a validated virus reduction neutralization test (VRNT), as previously described (*21**–**24*). In brief, serially diluted serum samples were mixed with wild-type DENV representing each of the 4 serotypes (DENV-1–4) before incubation with Vero cells. Cells were then fixed, stained with custom DENV-specific primary antibodies (GenScript, https://genscript.com) followed by Alexa Fluor 448-conjugated secondary antibodies (Invitrogen, https://invitrogen.com), and counted. The results were reported as a 60% VRNT (VRNT_60_) titer, the reciprocal of the serum dilution that reduced the number of infected cells by 60% compared with the virus control. We defined prior exposure to any DENV serotype (i.e., dengue seropositive at baseline) as having a VRNT_60_ titer against any of the 4 DENV serotypes above the lower limit of quantification (i.e., a VRNT_60_ titer of DENV-1 >18 or DENV-2 >15 or DENV-3 >12 or DENV-4 >13) at baseline (*21**–**24*).

### Active Surveillance of Participants in the DEN-03-IB Placebo Arm

We conducted active surveillance of the 5,947 participants who received placebo and were in the per-protocol population of DEN-03-IB over 64 months from start through the data cutoff for the primary analysis, February 22, 2016–July 13, 2021. We defined the data cutoff as the date at which all enrolled participants in either treatment group had completed >2 years of follow-up (22). We analyzed deidentified data to assess the epidemiology and natural history of dengue in Brazil. 

Site staff contacted participants by email, text message, or telephone at least monthly in the first 2 years and every 3 months afterwards to identify postvaccination adverse events and potential dengue cases. The sites could increase the frequency of follow-up contacts during times of localized outbreaks. In addition, we instructed participants in both treatment arms to seek out the trial team if they experienced a febrile illness or illness suspected to be dengue on the basis of clinical symptoms. We evaluated the presence and intensity of clinical symptoms during each unscheduled visit to assess suspected cases.

In cases of suspected dengue, a blood sample was collected, preferably within 9 days of symptom onset, for virologic confirmation. We extracted total RNA using QIAamp Viral RNA Mini Kit (QIAGEN, https://qiagen.com) before conducting reverse transcription PCR (RT-PCR) assays in a central laboratory. We made virologic confirmation of wild-type DENV and serotype determinations using a validated fourplex RT-PCR assay as previously described (*25*). In brief, we reversed transcribed and amplified total isolated RNA using DENV serotype–specific primer/probe sets targeting each of the 4 DENV serotypes (DENV-1–4). In addition, we tested samples separately for the presence of CHIKV and ZIKV viral RNA using previously described singleplex RT-PCRs with primer/probe sets targeting CHIKV or ZIKV (*26*, *27*).

### National Data

SINAN is a passive surveillance system that collects information from healthcare providers and institutions throughout Brazil on nationally notifiable diseases (*17*). Dengue cases in SINAN were defined by 2009 WHO diagnostic criteria (*28*) and confirmed through laboratory testing. In addition, healthcare providers reported basic demographic data (sex, age, race/ethnicity, and geographic location) from each dengue case (suspected, probable or confirmed) to SINAN. We pulled laboratory-confirmed dengue cases (confirmed by RT-PCR or IgM) from SINAN for the municipalities and time that DEN-03-IB took place. We disaggregated census data from the Instituto Brasileiro de Geografia e Estatística for the 15 municipalities that contained >1 of the 16 study sites.

### Expansion Factors

We determined expansion factors (EF) as the ratio of VCD incidence from active surveillance of the placebo arm of DEN-03-IB per 100,000 person-years divided by laboratory-confirmed dengue incidence from the national passive surveillance system, SINAN, per 100,000 population, as described previously (*19*). We defined VCD incidence from active surveillance as the number of virologically confirmed cases over person-years of follow-up, determined weekly as the cumulative time that each participant contributed during active surveillance over the duration of the study, and in 2019 during an epidemic year. Given the large age groups (2–59 years of age) included in DEN-03-IB, we calculated EF for the entire population of the 15 municipalities for interpretation feasibility. We calculated laboratory-confirmed dengue incidence from passive surveillance by dividing laboratory-confirmed dengue cases using RT-PCR or IgM by the municipality population based on census data (*19*). An EF >1 indicates potential underreporting by SINAN; an EF <1 indicates potential underreporting by active follow-up.

### Statistical Analyses

We summarized baseline demographics of all participants enrolled in the phase 3 trial (n=16,235) (Table 1). Covariates included age group at enrollment (2–6, 7–17, and 18–59 years of age), biologic sex (M/F), race (Pardo [multiracial Brazilian], White, Black, Asian, and Indigenous or Other), geographic region in Brazil (North, Northeast, Central-West, South, Southeast), prior exposure to any DENV serotype (yes/no) and a self-reported history of yellow fever vaccination before study enrollment (yes/no).

**Table 1:** Baseline demographics for participants enrolled in large phase 3 efficacy trial of a dengue vaccine, Brazil, 2016–2021*

Characteristic	Vaccine recipients	Placebo recipients
Total participants	10,259	5,976
Sex		
F	5,555 (54.1)	3,216 (53.8)
M	4,704 (45.9)	2,760 (46.2)
Age group, y		
2–6	3,337 (32.5)	1,679 (28.1)
7–17	3,376 (32.9)	1,771 (29.6)
18–59	3,546 (34.6)	2,526 (42.3)
Median age, y (range)	11 (2–59)	14 (2–59)
Race		
Pardo†	7,017 (68.4)	4,036 (67.5)
White	2,410 (23.5)	1,402 (23.5)
Black	655 (6.4)	408 (6.8)
Asian	149 (1.5)	114 (1.9)
Indigenous or other	28 (0.3)	16 (0.3)
Prior exposure to DENV, any serotype‡		
Y	5,009 (48.8)	3,041 (50.9)
N	4,855 (47.3)	2,700 (45.2)
Unknown or missing§	395 (3.9)	235 (3.9)
History of yellow fever vaccination		
Y	10,259 (100)	5,976 (100)
N	0	0

*Values are no. (%) except as indicated. Participants were from the DEN-03-IB trial of a live attenuated tetravalent dengue vaccine, Butantan-DV. DENV, dengue virus. †Multiracial Brazilian. ‡Prior exposure to DENV is defined as baseline 60% virus reduction neutralization test titer to any of the 4 DENV serotypes above the assay limit of detection. §Unknown result indicates missing test result, not consented sample, or participants who were not tested for DENV-3. Missing result indicates nonconsented participants for serology testing or participants who were not tested for other reasons.

We calculated estimates of incidence of virologically confirmed dengue, chikungunya, or Zika only among placebo recipients in the per-protocol population (n = 5,947) using a person-time approach. We defined incidence rate as cases per 100 person-years. We counted participants who experienced recurrent infections by >1 DENV serotype once in the total incidence and separately by serotype for the serotype-specific incidence. We determined incidence by age group according to participant age at the time of the event. We summarized incidence rates by demographic subgroup, baseline dengue serostatus for those with a known serostatus, and by study year. We calculated 95% CI for incidence rates using Poisson exact (*29*). 

We estimated EFs for each municipality to understand the degree of underreporting of dengue illness in the national passive surveillance system (*30*). We calculated 95% CI for EFs using Clopper-Pearson exact method (*31*). We calculated EFs and 95% CI during the entire follow-up study period (2016–2021) and during an outbreak year (2019). 

We summarized the frequency and distribution of clinical signs and symptoms associated with virologically confirmed dengue-associated or chikungunya-associated illness among placebo recipients and calculated 95% CIs using Clopper-Pearson exact method (*31*). We performed statistical analyses using SAS software version 9.4 (SAS Institute, Inc., https://www.sas.com).

## Results

### Participant Demographics and Baseline Serostatus

A total of 16,235 participants underwent randomization and received Butantan-DV (n = 10,259) or placebo (n = 5,976) during February 2016–July 2019. The baseline characteristics were comparable between the 2 arms; all participants had a history of previous yellow fever vaccination and approximately half of participants did not have evidence of prior dengue exposure before vaccination (Table 1). Of the 16,235 participants, 15,605 (96.1%) had a known dengue serostatus at baseline; 9,864 in the Butantan-DV arm and 5,741 in the placebo arm (Table 2). At baseline, most children ages 2–6 years of age (81.0%) and participants in the South region (91.8%) were DENV-naive. Although DENV seropositivity increased with age, 26.9% of adults (18–59 years old) were seronegative at baseline (Table 2).

**Table 2:** Dengue serostatus at baseline for participants enrolled in large phase 3 efficacy trial of a dengue vaccine trial, Brazil, 2016–2021*

Characteristic	Seropositive, no. (%)	Seronegative, no. (%)
Participants with a known baseline dengue serostatus	8,050 (49.6)	7,555 (46.5)
Sex		
F	4,493 (51.2)	3,910 (44.6)
M	3,557 (47.7)	3,645 (48.8)
Age at enrollment, y		
2–6	870 (17.3)	4,061 (81.0)
7–17	3,120 (60.6)	1,860 (36.1)
18–59	4,060 (66.9)	1,634 (26.9)
Race		
Pardo†	5,685 (51.4)	4,942 (44.7)
White	1,693 (44.4)	1,991 (52.2)
Black	514 (48.4)	492 (46.3)
Asian	133 (50.6)	115 (43.7)
Indigenous or other	25 (56.8)	15 (34.1)
Geographic region		
North	2,531 (51.1)	2,154 (43.5)
Northeast	2,591 (57.1)	1,766 (38.9)
Center-West	1,489 (52.9)	1,243 (44.2)
South	28 (5.7)	449 (91.8)
Southeast	1,411 (41.0)	1,943 (56.5)

*Participants were from the DEN-03-IB trial of a live attenuated tetravalent dengue vaccine, Butantan-DV. Prior exposure (i.e., dengue seropositive) is defined as having a baseline 60% virus reduction neutralization test titer to any of the 4 dengue virus serotypes above the assay limit of detection. †Multiracial Brazilian.

### Incidence of VCD

**Figure 1:**
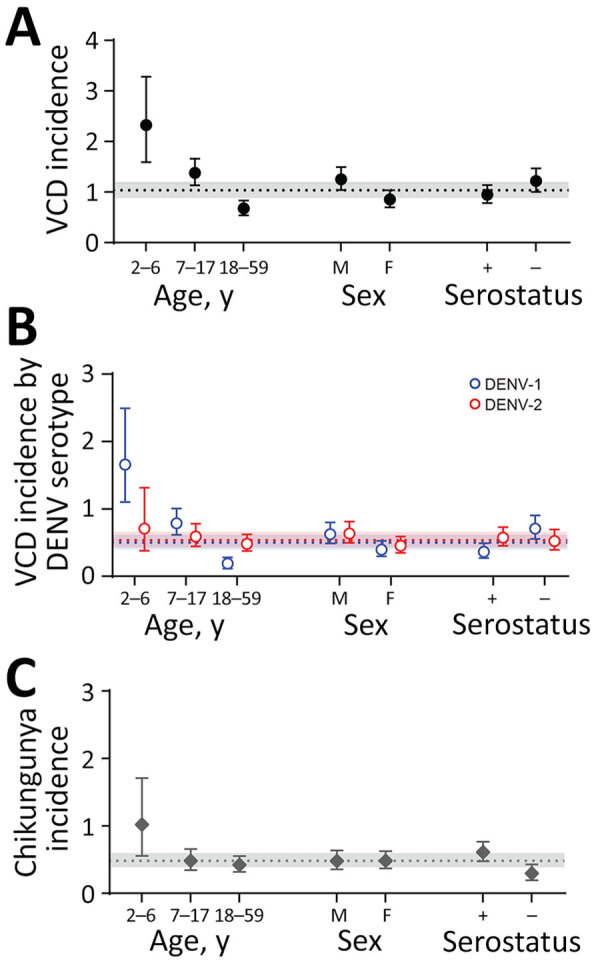
VCD and laboratory-confirmed chikungunya incidence by demographic characteristics, Brazil, 2016–2021. Incidence per 100 person-years of VCD is shown regardless of serotype (A), VCD by DENV serotypes DENV-1 and DENV-2 (B), and laboratory-confirmed chikungunya (C) among participants in the placebo arm of the DEN-03-IB trial of a live attenuated tetravalent dengue vaccine, Butantan-DV, by demographic characteristic (age at event, sex, and baseline dengue serostatus). Points represent incidence estimates and error bars represent 95% CIs. Dashed lines represent the overall incidence per 100 person-years and shaded areas around the dashed lines represent 95% CIs for the overall incidence estimates. Prior exposure (i.e., dengue seropositive) is defined as having a baseline 60% virus reduction neutralization test titer to any of the 4 DENV serotypes above the assay limit of detection. +, positive; –, negative; DENV, dengue virus; VCD, virologically confirmed dengue.

**Figure 2:**
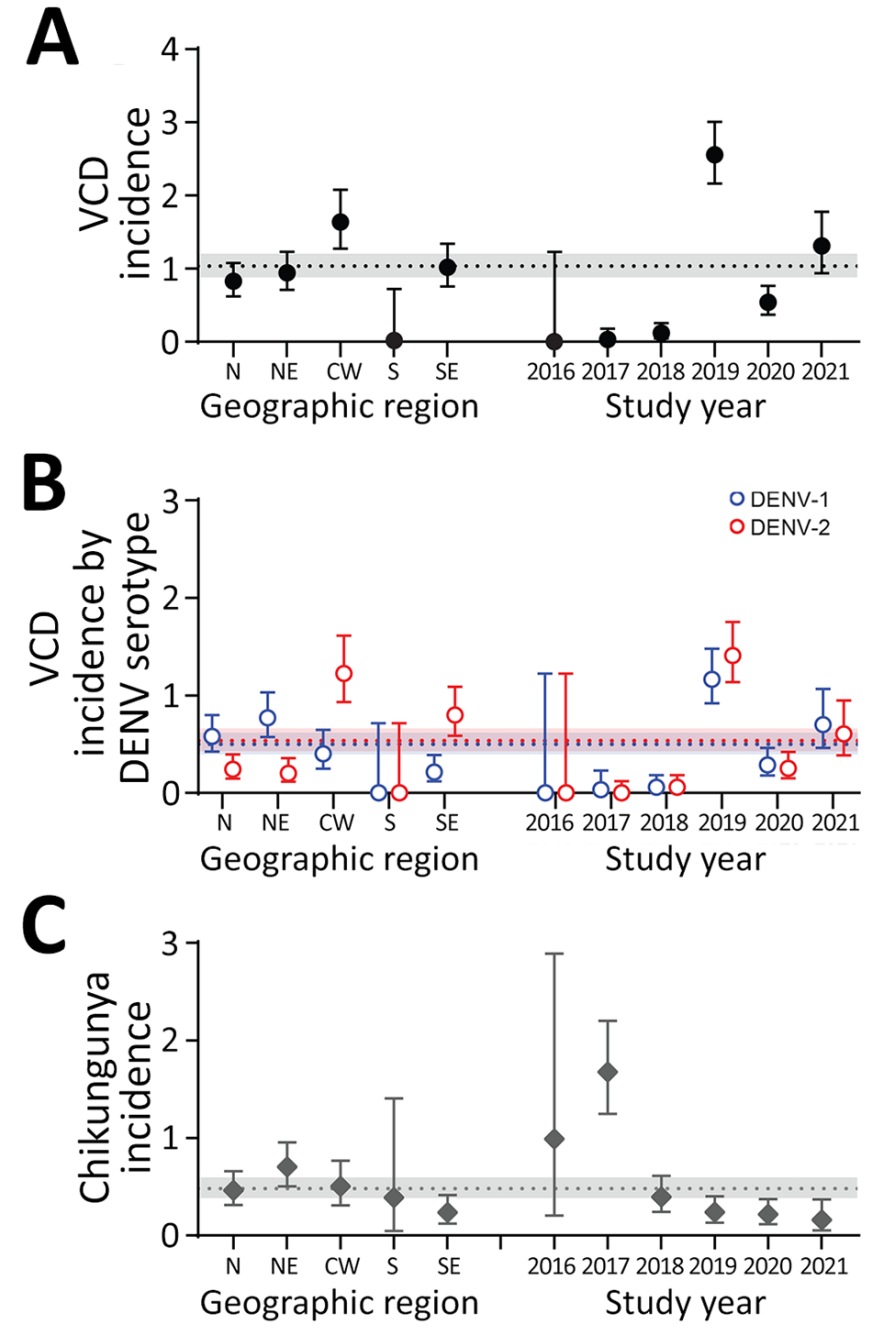
VCD and laboratory confirmed chikungunya incidence by temporospatial characteristics, Brazil, 2016–2021. Incidence per 100 person-years of VCD is shown regardless of serotype (A), VCD by DENV serotypes DENV-1 and DENV-2 (B), and laboratory-confirmed chikungunya (C) among participants in the placebo arm of the DEN-03-IB trial of a live attenuated tetravalent dengue vaccine, Butantan-DV, by temporospatial characteristic (geographic region and study year). Points represent incidence estimates and error bars represent 95% CIs. Dashed lines represent the overall incidence per 100 person-years and shaded areas around the dashed lines represent 95% CIs for the overall incidence estimates. CW, Center-West; DENV, dengue virus; N, North; NE, Northeast; S, South; SE, Southeast; VCD, virologically confirmed dengue.

Participants had completed >2 years of follow-up by the data cutoff (July 13, 2021). In total, 5,947 participants in the placebo arm had contributed to 22,028 person-years of follow-up. During follow-up, 228 cases of VCD from any DENV serotype were reported after Day 28 (incidence rate = 1.04/100 person-years), accounting for 1.71% of fever episodes (Appendix Table 1). Children 2–6 years of age had the highest rate of VCD (2.33/100 person-years) compared with older age groups (1.38/100 person-years for the 7–17 group and 0.68/100 person-years for the 18–59 group) (Figure 1). The Center-West region had the highest incidence (1.64/100 person-years); VCD was not observed in the South (Figure 2). VCD incidence peaked in 2019 (148 cases; 2.56/100 person-years).

We observed VCD cases resulting from DENV-1 (n = 111; 0.50/100 person-years) and DENV-2 (n = 119; 0.54/100 person-years) among participants in the placebo arm (Appendix Table 2); we did not observe cases of VCD resulting from DENV-3 or DENV-4 in the trial by the cutoff date. Two participants had dual infections of DENV-1 and DENV-2; in 1 participant they were concurrent and in the other, sequential. The Center-West and Southeast regions had a higher rate of DENV-2 (1.3/100 person-years in Center-West and 0.80/100 person-years in Southeast) compared with the North and Northeast, which were dominated by DENV-1 (0.58/100 person-years in the North region and 0.77/100 person-years in Northeast). We also observed differences in DENV serotype-specific incidence by age groups; DENV-1 incidence was higher in the 2–6 age group, DENV-2 was higher in the 18–59 age group, and both DENV serotypes had comparable incidence rates in the 7–17 age group.

### Estimating the Degree of Passive Surveillance System Underreporting for Dengue

**Figure 3:**
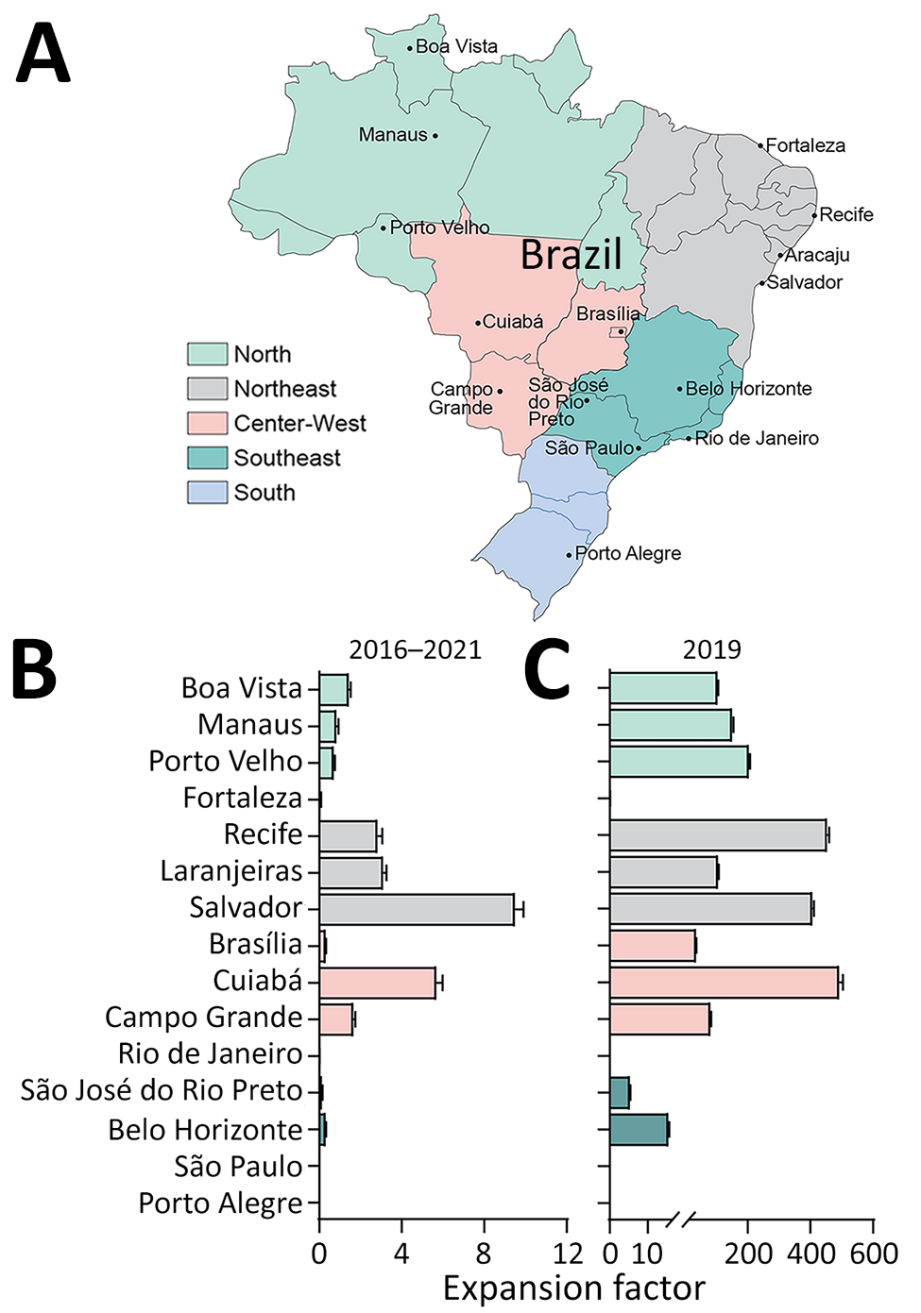
Locations of DEN-03-IB clinical study sites and expansion factors by municipality, Brazil, 2016–2021. A) Locations of clinical sites that enrolled participants in the DEN-03-IB phase 3 study of a live attenuated tetravalent dengue vaccine, Butantan-DV, in the 5 geographic regions (North, Northeast, Center-West, Southeast, South). B, C) Expansion factors and 95% CIs by municipality for the entire study period 2016–2021 (B) and during an outbreak year, 2019 (C). Note that 2 clinical sites were located in São Paulo and that Laranjeiras was the municipality closest to the clinical site in Aracaju.

Underreporting in the national passive surveillance system, SINAN, varied by municipality and over time (Figure 3). By municipality, considering the entire study period (2016–2021), EF range was <1 to 9.5 (Table 3). When considering only 2019, the year in which a major dengue outbreak occurred in Brazil (*10*, *32*, *33*), the EF range by municipality was 0–492.9 (Table 4).

**Table 3:** Incidence rates of virologically confirmed dengue and expansion factors, by municipality and state, Brazil, 2016–2021*

Municipality	DEN-03-IB incidence rate (95% CI)	SINAN incidence rate (95% CI)	Expansion factor (95% CI)†
São Paulo, SP‡	0 (0–414.4)	978.0 (972.5–983.5)	0 (0.0–0.004)
São José do Rio Preto, SP	2,069.1 (1,385.7–2,971.6)	14,134.9 (14,027.5–14,242.9)	0.15 (0.14–0.15)
Belo Horizonte, MG	1,249.2 (763.1–1,929.3)	3,951.4 (3,927.0–3,976.0)	0.32 (0.30–0.33)
Rio de Janeiro, RJ	0 (0–625.4)	283.6 (279.6–287.6)	0 (0–0.013)
Laranjeiras, SE§	1,576.2 (1,078.1–2,225.1)	507.8 (430.8–594.6)	3.1 (3.0–3.3)
Recife, PE	673.4 (336.1–1,204.8)	237.9 (230.6–245.5)	2.8 (2.6–3.1)
Fortaleza, CE	53.4 (1.4–297.5)	834.5 (823.6–845.4)	0.06 (0.05–0.08)
Salvador, BA	2,170.0 (1,083.3–3,882.8)	228.6 (223.1–234.1)	9.5 (9.1–9.9)
Manaus, AM	444.1 (213.0–816.8)	527.0 (517.6–536. 6)	0.84 (0.77–0.93)
Boa Vista, RR	1,214.5 (800.4–1,767.0)	846.8 (819.7–874.5)	1.4 (1.4–1.5)
Porto Velho, RO	786.5 (458.2–1,259.2)	1,110.1 (1,082.4–1,138.3)	0.71 (0.66–0.76)
Brasília, DF	1,480.9 (948.8–2,203.4)	4,661.7 (4,637.7–4,685.8)	0.32 (0.30–0.33)
Cuiabá, MT	1,494.4 (984.8–2,174.3)	263.0 (250.4–276.0)	5.7 (5.4–6.0)
Campo Grande, MS	2,030.7 (1,183.0–3,251.4)	1,219.4 (1,197.0–1,242.3)	1.7 (1.6–1.7)
Porto Alegre, RS	0 (0–705.7)	504.1 (492.8–515.6)	0 (0–0.007)

*Incidence rates of virologically confirmed dengue in the placebo arm of the DEN-03-IB trial of a live attenuated tetravalent dengue vaccine, Butantan-DV (cases/100,000 person-years), or dengue confirmed cases in SINAN (cases/100,000 population). AM, Amazonas; BA, Bahia; CE, Ceará; DF, Distrito Federal; MG, Minas Gerais; MS, Mato Grosso do Sul; MT, Mato Grosso; PE, Pernambuco; RJ, Rio de Janeiro; RO, Rondônia; RR, Roraima; RS, Rio Grande do Sul; SE, Sergipe; SINAN, Sistema de Informação de Agravos de Notificação; SP, São Paulo. †Expansion factor is the ratio of virologically confirmed dengue cases from active surveillance of the placebo arm of DEN-03-IB per 100,000 person-years over dengue confirmed cases from the national passive surveillance system, SINAN, per 100,000 population. A value >1 would indicate potential underreporting by SINAN, whereas a value <1 is indicative of potential underreporting by active follow-up. ‡Two DEN-03-IB clinical sites were located in São Paulo. §Used for the municipality closest to the Aracaju, SE, clinical site.

**Table 4:** Incidence rates of virologically confirmed dengue and expansion factors, by municipality and state, during the 2019 outbreak, Brazil*

Municipality	DEN-03-IB incidence rate (95% CI)	SINAN incidence rate (95% CI)	Expansion factor (95% CI)†
São Paulo, SP‡	0 (0.0–50,500.9)	235.9 (233.2–238.7)	0 (0.0–0.016)
São José do Rio Preto, SP	20,875.7 (11,412.9–35,025.9)	3,886.7 (3,830.2–3,944.1)	5.4 (5.3–5.4)
Belo Horizonte, MG	23,789.9 (14,323.1–37,150.9)	1,537.0 (1,521.7–1,552.4)	15.5 (15.3–15.7)
Rio de Janeiro, RJ	0 (0–83,895.6)	88.2 (86.0–90.5)	0 (0.0–0.042)
Laranjeiras, SE§	20,562.3 (14,064.6–29,027.8)	194.5 (147.7–251.4)	105.7 (104.3–107.2)
Recife, PE	25,657.6 (11,732.3–48,706.1)	56.5 (52.9–60.3)	454.0 (448.5–459.6)
Fortaleza, CE	0 (0–6,348.0)	57.7 (54.9–60.7)	0 (0.0–0.064)
Salvador, BA	33,913.6 (14,641.5–66,823.4)	83.3 (80.0–86.7)	407.1 (402.7–411.4)
Manaus, AM	7,180.1 (1,480.7–20,983.2)	47.6 (44.8–50.6)	150.8 (147.4–154.4)
Boa Vista, RR	20,652.3 (13,091.8–30,988.6)	197.9 (184.3–212.2)	104.4 (102.9–105.8)
Porto Velho, RO	14,922.4 (5,476.2–32,479.7)	73.1 (66.0–80.7)	204.2 (200.9–207.5)
Brasília, DF	22,474.3 (12,846.0–36,496.9)	625.6 (616.7–634.5)	35.9 (35.5–36.4)
Cuiabá, MT	7,557.4 (191.3–42,107.2)	15.3 (12.4–18.8)	492.9 (481.9–504.2)
Campo Grande, MS	21,297.4 (12,173.3–34,585.6)	259.9 (249.5–270.7)	81.9 (80.8–83.0)
Porto Alegre, RS	0 (0–26,450.0)	77.4 (73.0–82.0)	0 (0–0.048)

*Incidence rates of virologically confirmed dengue in the placebo arm of the DEN-03-IB trial of a live attenuated tetravalent dengue vaccine, Butantan-DV (cases/100,000 person-years), or dengue confirmed cases in SINAN (cases/100,000 population); AM, Amazonas; BA, Bahia; CE, Ceará; DF, Distrito Federal; MG, Minas Gerais; MS, Mato Grosso do Sul; MT, Mato Grosso; PE, Pernambuco; RJ, Rio de Janeiro; RO, Rondônia; RR, Roraima; RS, Rio Grande do Sul; SE, Sergipe; SINAN, Sistema de Informação de Agravos de Notificação; SP, São Paulo. †Expansion factor is the ratio of virologically confirmed dengue cases from active surveillance of the placebo arm of DEN-03-IB per 100,000 person-years over dengue confirmed cases from the national passive surveillance system, SINAN, per 100,000 population. A value >1 would indicate potential underreporting by SINAN, whereas a value <1 is indicative of potential underreporting by active follow-up. ‡Two DEN-03-IB clinical sites were located in São Paulo. §Used for the municipality closest to the Aracaju, SE, clinical site.

### Incidence of Other Laboratory-Confirmed Arboviral-Associated Illness

During the study period, 3 laboratory-confirmed Zika episodes (incidence rate 0.01/100 person-years) and 106 laboratory-confirmed chikungunya cases (incidence rate 0.48/100 person-years) were captured. We observed the highest incidence of laboratory-confirmed chikungunya in the 2–6 age group (1.02/100 person-years) spread out across the geographic locations; the Northeast region had the highest rates (0.70/100 person-years). Furthermore, we observed most laboratory-confirmed chikungunya cases (n = 54) in 2016 (0.99/100 person-years) and 2017 (1.68/100 person-years), the 2 years with the lowest rates of VCD during the study (Figure 2).

**Figure 4:**
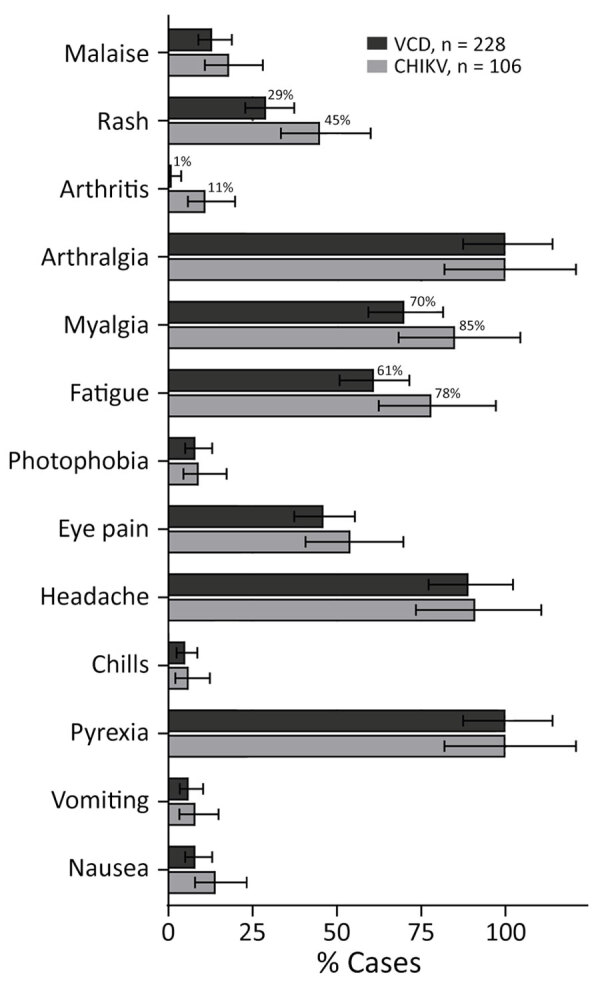
Clinical manifestations of VCD and CHIKV among participants in the placebo arm of the DEN-03-IB trial of a live attenuated tetravalent dengue vaccine, Butantan-DV, Brazil, 2016–2021. Bars represent percentages of participants with each symptom; error bars represent 95% CIs. Numeric percentages are indicated for symptoms with >10% difference between VCD and CHIKV cases. VCD, virologically confirmed dengue; CHIKV, laboratory-confirmed chikungunya.

The clinical manifestations of VCD and chikungunya cases during the follow-up were generally comparable; arthralgia, pyrexia, and headache were the most commonly reported symptoms among either VCD or chikungunya cases (Figure 4). Arthritis was more commonly reported among chikungunya cases (11%) than VCD cases (1%).

## Discussion

We used active surveillance from a phase 3 dengue vaccine efficacy trial (DEN-03-IB) to better understand dengue incidence in Brazil during 2016–2021. Dengue incidence was generally higher in our study than estimates reported in the national passive surveillance system but varied by municipality and study year. Nonspecific clinical manifestations overlapping with other circulating arboviruses, as we observed with laboratory-confirmed chikungunya in this study, may further contribute to underreporting of dengue. 

We used data from ≈6,000 participants 2–59 years of age who were enrolled in the placebo arm of the study to assess the incidence of dengue. Nearly a third of the participants in DEN-03-IB were adults (18–59 years of age); a large proportion of whom (26.9%) were dengue seronegative at baseline. Furthermore, active surveillance ensured that even mild cases of dengue were detected and distinguished from other arboviruses (ZIKV and CHIKV). Although the follow-up spanned multiple seasons (2016–2021), we detected only DENV-1 and DENV-2 in the study; we did not observe cases of VCD caused by DENV-3 or DENV-4. The absence of DENV-3 and DENV-4 reflects the reported circulation of DENV in Brazil during the period in which the trial was conducted (*10*). We observed regional and age differences in serotype-specific incidence for DENV-1 and DENV-2, suggesting previous population exposure histories may affect DENV serotype circulation. Consistent with previous reports that used passive surveillance (*34*–*41*), we found that the highest burden of dengue occurred in the Central-West region. Furthermore, in our study, children 2–6 years of age had the highest incidence of laboratory-confirmed dengue and chikungunya compared with the older age subgroups.

Placebo cohorts from phase 3 efficacy trials are a valuable data source to characterize dengue epidemiology within a country. Our findings add to previous results from other dengue vaccine phase 3 trials conducted in Brazil and provide report of active surveillance among adults (>18 years of age). Although those datasets are complementary, between-study comparisons are challenging because of differences in participant demographics, study site locations, and the period of follow-up. CYD15 was a phase 3 trial of the tetravalent dengue vaccine CYD-TDV (Dengvaxia; Sanofi, https://www.sanofi.com) conducted in 5 countries across 22 sites, including 5 sites in Brazil, during June 2011–April 2014 (*42*). In CYD15, overall VCD incidence was 3.5/100 person-years among 1,177 placebo recipients 9–16 years of age in Brazil (*43*), higher than what we observed in the DEN-03-IB trial from 2016–2021 (1.04/100 person-years). TIDES was a phase 3 trial of the tetravalent dengue vaccine TAK-003 (Qdenga; Takeda, https://www.takeda.com) conducted at 26 sites in 8 countries, including 4 sites in Brazil during September 2016–December 2021 (*44**,* *45*, *46*). In this similar timeframe, VCD incidence among 560 participants 4–16 years of age in Brazil (0.9/100 person-years) was generally comparable to rates observed in DEN-03-IB; incidence peaked in 2016 (caused by DENV-1) and 2019 (caused by DENV-2), consistent with DEN-03-IB. Still, the differences between incidence rates across trials could be attributed to differences by age or underlying serostatus of the study population and emphasize the variability and unpredictability of dengue epidemiology.

Previous studies of dengue epidemiology in Brazil have relied on data from SINAN, the national passive surveillance system. However, incidence rates from passive surveillance are likely underestimating the true burden of dengue because of the subclinical nature of dengue, particularly with the first infection, and because the clinical manifestation of dengue is similar to that of other arboviral diseases, including chikungunya and Zika. In addition, laboratory confirmation practices vary across healthcare institutions. We observed a large degree of underreporting in the national passive surveillance system (ratio of DEN-03-IB/SINAN>1), particularly during the dengue outbreak in 2019 (*10*,*32*,*33*). The increased underreporting in 2019 was likely caused by differences in surveillance practices during an outbreak, when the volume of cases overwhelmed testing and reporting systems; it is more challenging to conduct laboratory confirmation on suspected cases in those conditions (*47*). We also observed underreporting in the active surveillance (ratio of DEN-03-IB/SINAN<1) in some sites, particularly across the full study period of 2016–2021. A possible cause of the underreporting in active surveillance could be our limited ability to calculate EF from only a subset of the population, because clinical sites do not necessarily represent the entire municipality. EFs estimated from CYD15 using similar methods indicated that dengue was underreported in Brazil during 2011–2014 at a national (EF = 26.7), state (EF = 16.9), and local level (EF = 19.4) (*19*). Although it is difficult to compare EF estimates that are highly dependent on data availability, including age stratification, case definitions, and temporospatial factors, those data support the finding that underreporting of dengue in passive surveillance systems remains an important issue (*19*).

Two other arboviruses, ZIKV and CHIKV, co-circulate with DENV in Brazil (*12*). ZIKV was first identified in Brazil in 2015, before the initiation of the DEN-03-IB vaccine trial (*16*); ZIKV caused a major outbreak that peaked in February 2016, just as trial enrollment began, and subsequently circulated at low levels. In our study, 3 episodes of ZIKV were confirmed in participants under active surveillance. A possible explanation for the rarity of Zika cases in DEN-03-IB is the limited number of participants under active surveillance during the Zika outbreak; the last study participant enrolled in 2019 (*22*). An outbreak of chikungunya peaked in 2017; we captured many cases in our study. The overall low specificity of symptoms between chikungunya and dengue among participants in this study represents a misclassification risk that could contribute to underreporting to SINAN. The detection of both CHIKV and ZIKV infections, albeit at lower rates than VCD, illustrates the co-circulation of arboviruses in Brazil and the potential for misclassification in syndromic surveillance systems. Those observations confirm the need to reinforce laboratory confirmation practices.

DEN-03-IB enrolled participants 2–59 years of age in predefined urban centers; generalizability to older adults, rural populations, or other geographic regions may be limited. Few participants were enrolled in the single clinical site located in the South region of Brazil, limiting the interpretation of dengue circulation in that region. Several factors can affect incidence estimations including follow-up, withdrawal, or differential healthcare seeking across time and sites, although we minimized those factors in the randomized controlled trial. Nevertheless, the rigorous and standardized surveillance we conducted across all sites strengthens the internal validity and comparability of our findings. 

EF calculations have inherent limitations, such as the comparison of active surveillance in healthy participants with passive surveillance data from the municipalities’ general population. Furthermore, given the limited number of observations at the geographic level in this study, we did not stratify EFs by age. Although EF methodology is not perfect, EF estimates can provide a sense of the magnitude of dengue underreporting.

In sum, dengue epidemiology is unpredictable and varies over time by geography, serotype and age, even within a country. A better understanding of its epidemiology would inform public health interventions aimed at the prevention and control of dengue worldwide. Clinical manifestations are nonspecific, and surveillance remains an important tool to monitor arbovirus epidemiology. Furthermore, the differences in national surveillance systems, their reporting and laboratory-confirmation practices, and variable clinical definitions limit the extrapolation of surveillance data between countries. Our findings have direct implications for public health decision making, including the implementation of vaccination strategies and the interpretation of surveillance systems data. Longitudinal active surveillance embedded within vaccine trials represents a valuable approach to refining estimates of disease burden and informing control strategies in endemic settings.

Appendix Additional information about dengue incidence, seroprevalence, and expansion factors from active surveillance, Brazil, 2016–2021.
