# Supplementary material for: Dengue Incidence, Seroprevalence, and Expansion Factors from Active Surveillance, Brazil, 2016–2021
Source: Emerg Infect Dis. 2026 Apr;32(4):573–83. doi: 10.3201/eid3204.250942 (PMC13094852; doi:10.3201/eid3204.250942)
Supplement: Appendix — Additional information about dengue incidence, seroprevalence, and expansion factors from active surveillance, Brazil, 2016–2021. [file 25-0942-Techapp-s1.pdf]

Article DOI: https://doi.org/10.3201/eid3204.250942

EID cannot ensure accessibility for supplementary materials supplied by authors. Readers who have difficulty accessing supplementary content should contact the authors for assistance.

Dengue Incidence, Seroprevalence, and Expansion Factors from Active Surveillance, Brazil, 2016–2021

Appendix

Appendix Table 1. Incidence of febrile episodes and VCD by demographic characteristics, yellow fever vaccination status and baseline serostatus among DEN-03-IB placebo recipients*

Characteristic	Person-years	Pyrexia episodes	Pyrexia episodes incidence (Pe/Py)×100 (95% CI)	VCD cases overall†	VCD incidence overall (VCD/Py)×100 (95% CI)	% of VCD among pyrexia episodes (95% CI)
Overall	22,028.4	13,297	60.363 (59.341–61.398)	228/5,946	1.035 (0.905–1.178)	1.715 (1.499–1.952)
Age at event						
2-6	1,376.3	1,812	131.657 (125.664–137.862)	32/516	2.325 (1.590–3.282)	1.766 (1.208–2.493)
7-17	8,052.7	6,784	84.245 (82.252–86.274)	111/2,382	1.378 (1.134–1.660)	1.636 (1.346–1.970)
18-65	12,599.4	4,701	37.311 (36.252–38.393)	85/3,048	0.675 (0.539–0.834)	1.808 (1.444–2.236)
Age at event, median (range)	18.0 (2–65)	13.0 (2–65)	-	13.0 (2–62)	-	-
Sex						
Female	12,045.9	7,053	58.551 (57.192–59.934)	103/3,200	0.855 (0.698–1.037)	1.460 (1.192–1.771)
Male	9,982.4	6,244	62.550 (61.008–64.121)	125/2,746	1.252 (1.042–1.492)	2.002 (1.666–2.385)
Geographic location‡						
North	6,523.4	5,268	80.755 (78.589–82.966)	54/1,713	0.828 (0.622–1.080)	1.025 (0.770–1.337)
Northeast	5,826.6	4,210	72.255 (70.089–74.471)	55/1,624	0.944 (0.711–1.229)	1.306 (0.984–1.700)
Center-West	4,147.9	1,864	44.938 (42.921–47.026)	68/1,132	1.639 (1.273–2.078)	3.648 (2.833–4.625)
South	522.2	166	31.789 (27.137–37.009)	0/136	0.000 (0.000–0.706)	0.000 (0.000–2.222)
Southeast	5,008.3	1,789	35.721 (34.084–37.415)	51/1,341	1.018 (0.758–1.339)	2.851 (2.123–3.748)
Baseline serostatus						
Seronegative	9,096.6	6,877	75.600 (73.823–77.408)	111/2,689	1.220 (1.004–1.469)	1.614 (1.328–1.944)
Seropositive	11,922.6	5,944	49.855 (48.595–51.139)	113/3,023	0.948 (0.781–1.139)	1.901 (1.567–2.286)
Unknown or missing	1,009.2	476	47.166 (43.024–51.600)	4/234	0.396 (0.108–1.015)	0.840 (0.229–2.152)
Year						
2016	303.7	77	25.354 (20.009–31.688)	0/1,364	0.000 (0.000–1.215)	0.000 (0.000–4.791)
2017	3,075.0	1641	53.366 (50.815–56.012)	1/4,321	0.033 (0.001–0.181)	0.061 (0.002–0.340)
2018	5,061.8	3,191	63.041 (60.872–65.267)	6/5,636	0.119 (0.043–0.258)	0.188 (0.069–0.409)
2019	5,783.5	4,675	80.833 (78.533–83.184)	148/5,946	2.559 (2.163–3.006)	3.166 (2.676–3.719)
2020	5,919.2	2,503	42.286 (40.646–43.976)	32/5,946	0.541 (0.370–0.763)	1.278 (0.874–1.805)
2021	3,130.1	1,210	38.657 (36.509–40.898)	41/5,946	1.310 (0.940–1.777)	3.388 (2.432–4.597)

*py, person-year; VCD, virologically confirmed dengue (RT-PCR) occurring more than 28 days after treatment through data cutoff on 13-JUL-2021.
†One subject was infected by two virus types sequentially; one subject was infected by two virus types simultaneously.
‡ Municipalities included in each region. (1) Northeast: C5-Laranjeiras; C6-Recife; C7-Fortaleza; C15-Salvador. (2) North: C8-Manaus; C9-Boa Vista; C11-Porto Velho. (3) Center-West: C10-Brasília; C12-Cuiabá; C13-Campo Grande. (4) South: C14-Porto Alegre. (5) Southeast: C1-ICHC, Sao Paulo; C2-Santa Casa; C3-Rio Preto, Sao Jose; C4-Belo Horizonte; C16-Rio de Janeiro.

Appendix Table 2. Incidence of VCD by serotype by demographic characteristics, geographic location, yellow fever vaccination status, baseline serostatus and year, among DEN-03-IB placebo recipients*

Characteristic	Person-years (DENV-1)	VCD episodes (DENV-1)	VCD (DENV-1) incidence (VCD/py)×100 (95% CI)	Person-years (DENV-2)	VCD episodes (DENV-2)	VCD (DENV-2) incidence (VCD/py)×100 (95% CI)	Overall number of VCD cases†
Overall	22,231.5	111/5,946	0.499 (0.411–0.601)	22,198.6	119/5,946	0.536 (0.444–0.641)	228/5,946
Age at event							
2-6	1,388.8	23/516	1.656 (1.050–2.485)	1,415.8	10/516	0.706 (0.339–1.299)	32/516
7-17	8,126.3	64/2,382	0.788 (0.607–1.006)	8,154.6	48/2,382	0.589 (0.434–0.780)	111/2,382
18-65	12,716.3	24/3,048	0.189 (0.121–0.281)	12,628.1	61/3,048	0.483 (0.369–0.620)	85/ 3,048
Age at event, median (range)	18.0 (2–65)	11.0 (2–61)	-	18.0 (2–65)	18.0 (3–62)	-	13.0 (2–62)
Sex							
Female	12,139.1	48/3,200	0.395 (0.292–0.524)	12,127.4	55/3,200	0.454 (0.342–0.590)	103/3,200
Male	10,092.4	63/2,746	0.624 (0.480–0.799)	10,071.2	64/2,746	0.635 (0.489–0.811)	125/2,746
Geographic location‡							
North	6,543.7	38/1,713	0.581 (0.411–0.797)	6,589.0	16/1,713	0.243 (0.139–0.394)	54/1,713
Northeast	5,843.5	45/1,624	0.770 (0.562–1.030)	5,913.3	12/1,624	0.203 (0.105–0.354)	55/1,624
Center-West	4,235.2	17/1,132	0.401 (0.234–0.643)	4,160.3	51/1,132	1.226 (0.913–1.612)	68/1,132
South	522.2	0/136	0.000 (0.000–0.706)	522.2	0/136	0.000 (0.000–0.706)	0/136
Southeast	5,086.8	11/1,341	0.216 (0.108–0.387)	5,013.7	40/1,341	0.798 (0.570–1.086)	51/1,341
Baseline serostatus							
Seronegative	9,176.8	65/2,689	0.708 (0.547–0.903)	9,193.5	48/2,689	0.522 (0.385–0.692)	111/2,689
Seropositive	12,044.2	44/3,023	0.365 (0.265–0.490)	11,994.1	69/3,023	0.575 (0.448–0.728)	113/3,023
Unknown or missing	1,010.5	2/234	0.198 (0.024–0.715)	1,011.0	2/234	0.198 (0.024–0.715)	4/234
Year							
2016	303.7	0/1,364	0.000 (0.000–1.215)	303.7	0/1,364	0.000 (0.000–1.215)	0/1,364
2017	3,075.0	1/4,321	0.033 (0.001–0.181)	3,075.6	0/4,321	0.000 (0.000–0.120)	1/4,321
2018	5,062.2	3/5,636	0.059 (0.012–0.173)	5,063.1	3/5,636	0.059 (0.012–0.173)	6/5,636
2019	5,835.6	68/5,946	1.165 (0.905–1.477)	5,812.5	82/5,946	1.411 (1.122–1.751)	148/5,946
2020	5,930.3	17/5,946	0.287 (0.167–0.459)	5,931.0	15/5,946	0.253 (0.142–0.417)	32/5,946
2021	3,135.7	22/5,946	0.702 (0.440, 1.062)	3,136.4	19/5,946	0.606 (0.365–0.946)	41/5,946

*py, person-year; VCD, virologically confirmed dengue (RT-PCR) occurring more than 28 days after treatment through data cutoff on 13-JUL-2021.

†One subject was infected by two virus types sequentially; one subject was infected by two virus types simultaneously.

‡ Municipalities included in each region. (1) Northeast: C5-Laranjeiras; C6-Recife; C7-Fortaleza; C15-Salvador. (2) North: C8-Manaus; C9-Boa Vista; C11-Porto Velho.(3) Center-West: C10-Brasília; C12-Cuiabá; C13-Campo Grande. (4) South: C14-Porto Alegre. (5) Southeast: C1-ICHC, Sao Paulo; C2-Santa Casa; C3-Rio Preto, Sao Jose; C4-Belo Horizonte; C16-Rio de Janeiro.
